# D2-CovidNet: A Deep Learning Model for COVID-19 Detection in Chest X-Ray Images

**DOI:** 10.1155/2021/9952109

**Published:** 2021-12-15

**Authors:** Xin Wang, Yiyang Hu, Yanhong Luo, Wei Wang

**Affiliations:** ^1^School of Computer and Communication Engineering, Changsha University of Science and Technology, Changsha 410114, China; ^2^Hunan Children's Hospital, Changsha 410000, China

## Abstract

Since the outbreak of Coronavirus disease 2019 (COVID-19), it has been spreading rapidly worldwide and has not yet been effectively controlled. Many researchers are studying novel Coronavirus pneumonia from chest X-ray images. In order to improve the detection accuracy, two modules sensitive to feature information, dual-path multiscale feature fusion module and dense depthwise separable convolution module, are proposed. Based on these two modules, a lightweight convolutional neural network model, D2-CovidNet, is designed to assist experts in diagnosing COVID-19 by identifying chest X-ray images. D2-CovidNet is tested on two public data sets, and its classification accuracy, precision, sensitivity, specificity, and *F*1-score are 94.56%, 95.14%, 94.02%, 96.61%, and 95.30%, respectively. Specifically, the precision, sensitivity, and specificity of the network for COVID-19 are 98.97%, 94.12%, and 99.84%, respectively. D2-CovidNet has fewer computation number and parameter number. Compared with other methods, D2-CovidNet can help diagnose COVID-19 more quickly and accurately.

## 1. Introduction

Coronavirus disease 2019 (COVID-19) caused by the 2019 novel Coronavirus (2019-nCoV) has spread all over the world in a very short time. The detection technology based on reverse transcription polymerase chain reaction (RT-PCR) is the most widely used method for diagnosing COVID-19. However, because its kits are limited and expensive, the diagnosis is time-consuming [1]. This approach has certain limitations in the diagnosis process and easy to get false negative result.

At present, the auxiliary diagnosis and treatment methods for COVID-19 mainly include chest X-ray and computed tomography (CT) [2]. Compared with CT, the structure and tissue of the lesion in the chest X-ray image are more obvious, and the X-ray device is more popular [3]. For radiologists, manual reading is a labor-intensive task that is time-consuming and error prone. Therefore, an effective and fast auxiliary detection method for COVID-19 could significantly reduce the pressure on medical staff and resource supply and reduce the risk of infection among medical staff.

In recent years, the application of deep learning methods in the field of computer vision has achieved good results, and the advantages of convolutional neural networks (CNNs) in feature extraction have been proven. For example, Wang et al. [4] proposed the Dense-MobileNet model, which can make full use of the output feature maps generated by previous convolution layers in dense blocks, so as to generate a large number of feature maps with fewer convolution kernels and reuse these feature. Mei et al. [5] used CNNs and traditional machine learning algorithm to quickly diagnose COVID-19-positive patients. Wang et al. [6] proposed and improved a deep learning approach with global average pooling (GAP) to classified colonoscopy polyp images for assisted diagnosis. Wang et al. [7] designed the channel feature weight extraction module (CFWE) according to the characteristics of chest X-ray image and proposed a new CFW-Net. Apostolopoulos and Mpesiana [8] used a CNN based on the transfer learning method to automatically detect X-ray images. Wang et al. [9] designed a COVID-19 network for detecting COVID-19 cases from chest X-ray images and investigated how interpretable methods could be used for prediction in an attempt to gain insight into the key factors associated with COVID-19 cases. Khan et al. [10] proposed a deep CNN model based on Xception-CoroNet and used a pretraining method based on the ImageNet dataset to identify COVID-19-positive chest X-ray images. Ozturk et al. [11] proposed a DarkCovidNet model with fewer parameters to automatically detect COVID-19-positive chest X-ray images.

CNNs have been widely used in computer vision tasks, such as image classification [12], target detection, and semantic segmentation [13]. Diagnosis accuracy is critical to preventing epidemics, but chest X-ray images have high feature similarity between classes and low intraclass feature variability, which could cause model deviation or overfitting and increase the difficulty of model identification of COVID-19. To solve the above problems, dual-path multiscale fusion (DPM) module and dense depthwise separable (DDS) module, which are sensitive to feature information are proposed. Based on these two modules, a new lightweight CNN model, D2-CovidNet, is designed for COVID-19 detectiong in X-ray images.

## 2. Architecture Design

### 2.1. DPM Module and DDS Module

**Figure 1:**
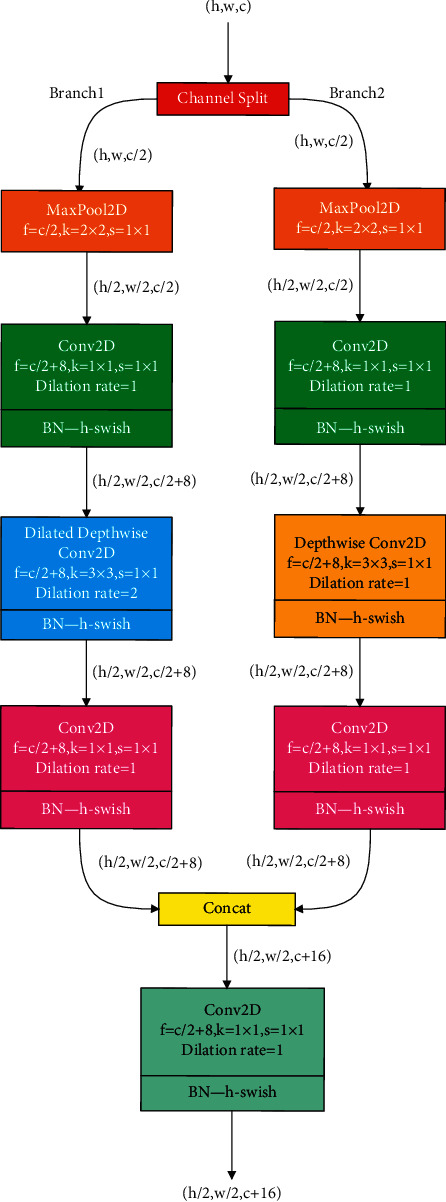
The structure of DPM module.

**Figure 2:**
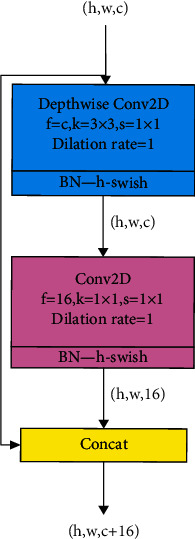
The structure of DDS module.

Due to the chest X-ray images with high similarity between categories and low intracategory variability, DPM module and DDS module are proposed. These two modules, as shown in Figures 1 and 2, have strong characterization capabilities and efficient computing abilities. In Figures 1 and 2, *h*, *w*, and *c* denote the height, width, and channels of the feature map, respectively. *k* is the kernel size, *s* is the strides, and *f* is the number of filters in the convolution. In these modules, “h-swish” is employed as the activation function, which can reduce the time delay and make the model suitable for mobile devices [14].

The DPM module contains 2 branches, and each branch contains 1 pooling layer and 3 convolutional layers. Maximum pooling layer with the filter size of 2 × 2 and step size of 2 × 2 is used as the pooling layer. Maximum pooling can ensure the invariance of the position and rotation of the features, which reduce the number of parameters of the model, thereby alleviating the problem of overfitting. The first 1 × 1 convolutional layer increases the channel number of the feature map for enriching the feature information. The second 1 × 1 convolutional layer is used to correlate the output feature maps with different channel information, which is depthwise convolution or dilated depthwise convolution, for example, depthwise convolution using convolutional filters with the dilation rate of 2. Since the dilated convolution [15] does not increase the number of parameters and the amount of computation, the complexity of the two branches is the same, making the model better suitable for dual-path-parallel computing. The features extracted by the convolutional layer close to the input contain detailed texture information, so the DPM module is used in the shallow layers of the network.

### 2.2. D2-CovidNet

**Figure 3:**
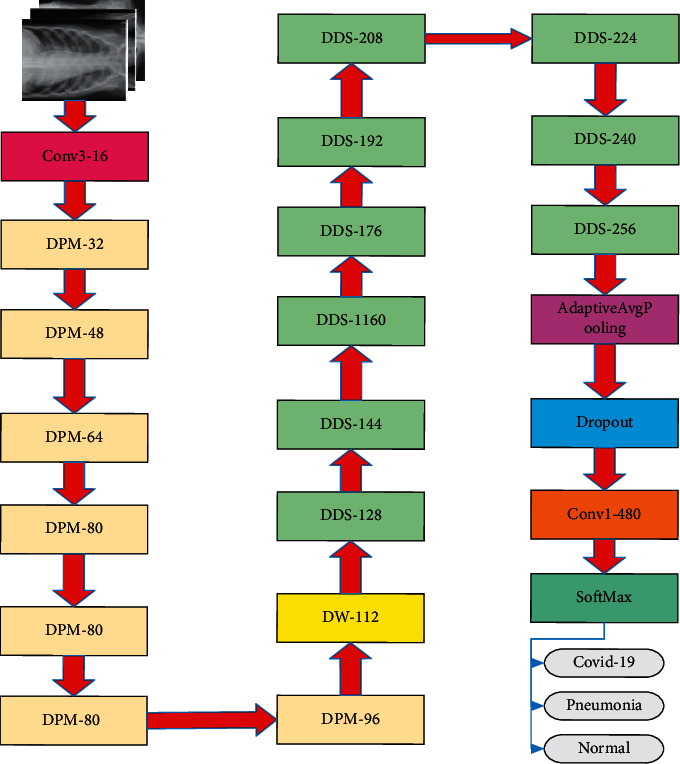
The structure of D2-CovidNet.

Based on DPM module and DDS module, a new lightweight CNN, D2-CovidNet, and an automatic detection approach for COVID-19 detection are proposed, as shown in Figure 3.

In Figure 3, the first layer contains a dilated convolution filter with an expansion rate of 2. Then, the DPM module is used for 5 times to halve the spatial dimension of the feature map for five times, reducing the size of the model. Next, the depthwise separable convolution layer (DW) [16] is used to enrich feature information, and the DDS module is used for 9 times to extract features to alleviate the disappearance of gradients. After the adaptive average pooling layer, the space size of the feature map becomes 1 × 1. To prevent overfitting, the obtained output feature map is subjected to a dropout layer with the drop rate of 0.2, and then a full connection layer is used to increase dimensionality of feature. Finally, the SoftMax layer is employed for classification.

### 2.3. Network Complexity

Amount of computation and the number of parameters are used to measure the complexity of the model. Introducing the dilated convolution with the expansion rate of 2 will not change the number of parameters and the amount of computation. The depthwise separable convolutional layer has fewer parameters and computations than the traditional convolutional layer, which can save memory and running time. Compared with serial connection, the dual-path structure can reduce the model size and the number of memory accesses.

**Figure 4:**
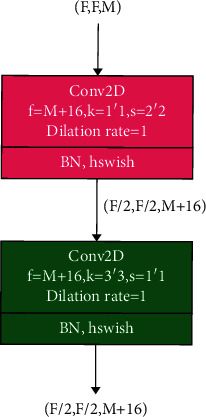
A ordinary convolutional module to achieve dimensional transformation.

For a given module, which contains two ordinary convolutional layers, as shown in Figure 4, the size of input feature map is *F* × *F* × *M* and the size of output feature map is (*F*/2) × (*F*/2) × (*M* + 16). In Figure 4, *F* × *F* means the spatial dimension of the input feature map and the channel of the input feature map *M* ≥ 16. Meanwhile, *f*, *k*, and *s* represent the number of convolution kernels, the size of convolution kernels, and the step size of convolution kernels, respectively. The number of parameters *P*_OC 1_ and the number of computations *F*_OC 1_ are as follows.


(1) $$\begin{array}{c} P_{\text{OC }1}\;=\;\left(M+16\right)\times M+{\left(M+16\right)}^{2}\times 9,\\ F_{\text{OC }1}=\;\left(M+16\right)\times M\times F^{2}+{\left(M+16\right)}^{2}\times {\left(\frac{F}{2}\right)}^{2}\times 9. \end{array}$$

The number of parameters *P*_DPM_ and computations *F*_DPM_ generated by the DPM module with dilated depthwise convolution of stride 1 is, respectively, shown as follows:

(2) $$\begin{array}{c} P_{\text{DPM}}=\;\left(\left(\frac{M}{2}+8\right)\times \frac{M}{2}+{\left(\frac{M}{2}+8\right)}^{2}\times 10\right)\times 2,\\ F_{\text{DPM}}=\;\left(\left(\frac{M}{2}+8\right)\times \frac{M}{2}+{\left(\frac{M}{2}+8\right)}^{2}\times 10\right)\times 2\times {\left(\frac{F}{2}\right)}^{2}. \end{array}$$

Therefore, compared with the module shown in Figure 4, the reduction in parameter Δ*P*_DPM_ and computation Δ*F*_DPM_ achieved by DPM module is shown as follows:

(3) $$\begin{array}{c} \Delta P_{\text{DPM}}=\;P_{\text{OC }1}-\;P_{\text{DPM}}=\;\frac{9}{2}\cdot M^{2}+136\cdot M+1024,\\ \Delta F_{\text{DPM}}=\;F_{\text{OC }1}-\;F_{\text{DPM}}=\;\frac{15}{8}\cdot M^{2}\cdot F^{2}+46\cdot M\cdot F^{2}+256\cdot F^{2}. \end{array}$$

The parameters of D2-CovidNet are shown in Table 1. The flops and parameters of some deep learning models are as shown in Table 2 (the least number of flops and the least number of parameters are bolded). According to Table 2, D2-CovidNet has the least number of computations and amount parameters among the networks above. D2-CovidNet is appropriately 262.90 times smaller than VGG19 in computations and 685.20 times smaller than VGG19 in parameters.

## 3. Experimental Results and Analysis

### 3.1. Dataset

**Figure 5:**
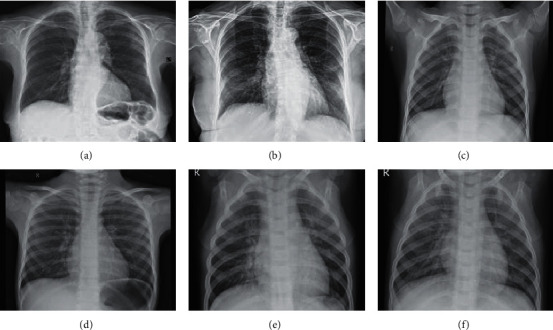
Cases of chest X-ray images: (a-b) COVID-19 images; (c-d) normal images; (e-f) other pneumonia images.

The chest X-ray images used in our experiments are from two open-source datasets. The common pneumonia and normal chest X-ray images are selected from the data set provided by Kaggle [26] (https://www.kaggle.com/paultimothymooney/chest-xray-pneumonia). It contains a total of 5863 chest X-rays images. 4265 chest X-ray images of pneumonia and 1575 normal chest X-ray images are selected from this dataset. The COVID-19 chest X-ray images used in our experiments are selected from the dataset (https://github.com/ieee8023/covid-chestxray-dataset) collected by Cohen et al. [27]. This dataset contains 790 chest X-ray images and CT images of infected patients with COVID-19 or other pneumonia. 412 chest X-ray images of with COVID-19 patients are selected from this dataset.  Figure 5 shows a partial example of various chest X-ray images from the data set used in the experiments. Finally, 102 COVID-19 X-ray images, 234 normal X-ray images, and 390 pneumonia X-ray images are randomly selected as the test set and the rest as the training set.

Chest X-ray image features have high interclass similarity and low intraclass variance, causing the model classification deviation or over fitting. According to the evaluation criteria adopted by most medical image classification models, accuracy, precision, specificity, sensitivity, and *F*1-score are used as performance indicators.

The formulas for these evaluation criteria are as follows:

(4) $$\begin{array}{c} \text{accuracy}=\frac{\text{TP}+\text{TN}}{\text{TP}+\text{TN}+\text{FP}+\text{FN}},\\ \text{precision}=\frac{\text{TP}}{\text{TP}+\text{FP}},\\ \text{sensitivity}=\frac{\text{TP}}{\text{TP}+\text{FN}},\\ \text{specificity}=\frac{\text{TN}}{\text{TN}+\text{FP}},\\ F1-\text{score}=\frac{2\times \text{TP}}{2\times \text{TP}+\text{FP}+\text{FN}}. \end{array}$$

Among these equations, TP represents true positive, FP represents false positive, FN represents false negative, and TN represents true negative.

### 3.2. Preprocessing and Parameter Setting

Image augmentation techniques and pretraining methods are adopted based on chest X-ray image datasets to overcome the lack of data. In the experiments, firstly, the chest X-ray images are resized to a fixed resolution of 224 × 224, randomly selected half of the training set images for 60-degree horizontal flipping and randomly selected half of the training set images for 45-degree vertical flipping. Then, the values of the brightness, the contrast, and the saturation of the chest X-ray images are changed uniformly 0.6–1.4 times. Therefore, the number of samples used for training is 5 times that of the training set through data enhancement technology, enhancing the generalization performance of the model.

The weight parameters of the convolutional layer of the experimental model are initialized by using Kaiming normal distribution [28]. The weight value in the batch normalization layer is 1, and the bias value is 0. The weight parameters in the full connection layer are initialized with a normal distribution with a mean value of 0 and a standard deviation of 0.01. We conducted 10 sets of experiments in the same configuration environment, including 9 sets of comparative experiments. The software platform and hardware environment of our experiment are shown in Table 3.

The initial learning rate (LR) of the experimental models is set to be 0.01. For each set, models are trained for 200 epochs. For the first 20 epochs, Adam optimizer [29] is adopted with betas between 0.9 and 0.999 to make the model converge quickly. In the last 120 epochs, the SGD optimizer [30] is used with a momentum of 0.9 and a weight decay of 5*e*−4 to find the optimal solution of the model. Meanwhile, the method of adjusting the learning rate is employed periodically. At the 50th epoch, the learning rate is set to 0.005. When epoch ∈ [60, 70, 80, 90, 110, 130, 150], the decay rate of the LR corresponds to [1/4, 1/8, 1/16, 1/32, 1/20, 1/40, 1/80, 1/100]. The “BatchSize” of the training set and test set is 32 and 16, respectively.

### 3.3. Recognition Results

**Figure 6:**
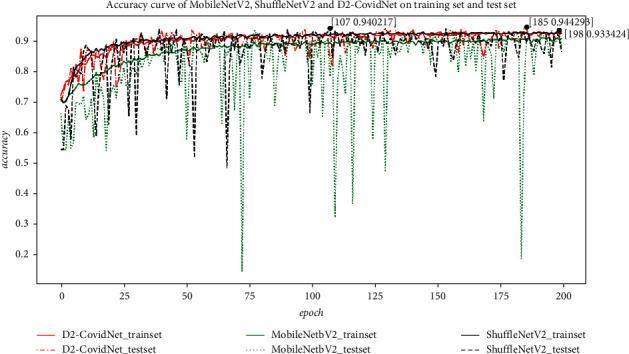
Accuracy curves of MobileNetV2, ShuffleNetV2, and D2-CovidNet on training set and test set.

**Figure 7:**
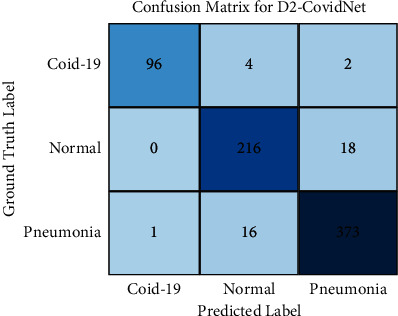
Confusion matrix of D2-CovidNet.

To reflect the lightweight and good recognition performance of our proposed model, MobileNetV2 and ShuffleNetV2 are used for comparative experiments. The accuracy curves of MobileNetV2, ShuffleNetV2, and D2-CovidNet are shown in Figure 6. The average accuracy of every 50 epochs and model complexity is shown in Table 4. The best performing model and model parameters are saved for each network model. It can be seen from Figure 7 that D2-CovidNet is more stable than MobileNetV2 and ShuffleNetV2 on the experimental data set. As can be seen from Table 3, the average accuracy rate of the last 10 epochs of D2-CovidNet is 2.22% higher than that of MobileNetV2 and 2.91% higher than that of ShuffleNetV2. However, the number of computations and parameters of D2-CovidNet is only 0.23 times and 0.09 times of MobileNetV2 and 0.49 times and 0.16 times of ShuffleNetV2, respectively. Thus, on the experimental data set, D2-CovidNet performs better than MobileNetV2 and ShuffleNetV2, and the complexity of D2-CovidNet is lower than that of MobileNetV2 and ShuffleNetV2.

To reflect the effectiveness of our model, other 7 CNN models are used to conduct comparative experiments on the expanded data set, including several traditional CNN models, such as VGG19, GoogleNet, ResNet50, and DenseNet121, and several lightweight CNN models, such as SqueezeNet1.0, MobileNet, and ShuffleNet. The accuracy, precision, sensitivity, specificity, and *F*1-score of the models are shown in Table 5 (the best results are bolded).

As can be seen from Tables 1, 4, and 5, D2-CovidNet has the lowest complexity and the best overall performance among these models. Table 5 shows that the classification accuracy, sensitivity, specificity, and *F*1-score of D2-CovidNet are 94.43%, 94.02%, 96.61%, and 95.30%, respectively. These values are higher than those of other models. Among the traditional CNN models, ResNet50 has the highest classification accuracy of 93.53%, but it is still 0.90% lower than D2-CovidNet, and its computation amount and parameter amount are 54.99 times and 112.10 times those of D2-CovidNet, respectively. From Table 5, among the lightweight CNN models, MobileNet has the highest classification accuracy rate of 88.53%, but it is 5.90% lower than D2-CovidNet. However, the computation amount and parameter amount of MobileNet are 7.87 times and 15.55 times those of D2-CovidNet, respectively. Different from the ShuffleNet unit and the inception structure in GoogleNet, the complexity of each branch in DPM module is the same, which can be better applied to dual-path-parallel computing. The DPM model implements different scales feature fusion to enhance model representation ability and improve model performance. Unlike DenseNet121, which used dense connections in the entire network, D2-CovidNet only used dense connections in the deeper layers of the network. This improves the utilization of feature maps and effectively alleviates the problem of gradient disappearance. Therefore, D2-CovidNet can effectively identify COVID-19 X-ray images and can be applied to mobile devices.

The features extracted by the convolutional layer near the input include detailed texture information such as contours, and the features extracted by the convolutional layer near the output include rich semantic information. Therefore, D2-CovidNet uses DPM module with strong characterization capabilities in the shallow layer of the network and DDS module that can realize feature reuse in the deep layer of the network. After many fine-tuning and experimental verification, D2-CovidNet in this article has higher recognition performance and lower computing overhead and is the most cost-effective choice among multiple configurations.

In order to further verify the necessity of using a specific number of different modules in the shallow and deep layers of the model, we conducted a more comprehensive ablation experiment. Two configurations were used in the ablation experiment: “F-DPM” and “F-DDS,” respectively. “F-DPM” is composed only of DPM modules, and “F-DDS” is composed only of DDS modules. The above two models are used to conduct experiments in the same experimental environment, and the experimental results are shown in Table 6.

It can be seen from Table 6 that the network performance using only the DPM module or the DDS module is not as good as D2-CovidNet. For a network model, it is not enough to have shallow information extraction capabilities or deep information extraction capabilities. Only when these two capabilities are used in a reasonable combination can the feature extraction capabilities be maximized, which also verifies the superiority of D2-CovidNet.

In addition, some deep learning methods for automatic detection of COVID-19 chest X-ray images are compared with D2-CovidNet, as shown in Table 6 (the best results are bolded). In Table 6, among the 4 comparison models, DarkCovidNet [11] has the least number of parameters, but it is still 5.50 times that of D2-CovidNet, and its classification accuracy is 7.41% lower than D2-CovidNet. CoroNet [10] has the highest accuracy, which is only 0.16% higher than D2-CovidNet, but its parameter amount is 165.00 times that of D2-CovidNet. For such a large difference, an intuitive explanation is that deep separable convolution is used many times in our network. Considering the network performance and complexity, D2-CovidNet is a recommended intelligent method for identifying chest X-ray images of COVID-19.

Figure 7 shows the confusion matrix of D2-CovidNet on test set, and Table 7 shows the specific performance of D2-CovidNet on various performance indicators.

It can be seen from Table 8 that the average accuracy, average sensitivity, and average specificity of D2-CovidNet are all higher than 90%, which are 95.14%, 94.02%, and 96.16%, respectively. Especially, the accuracy, sensitivity, and specificity of D2-CovidNet's recognition of COVID-19 are 98.97%, 94.12%, and 99.84%, respectively. H. Wong et al. mentioned that the baseline sensitivity of COVID-19 chest X-ray images was 69% [31], so our proposed D2-CovidNet can effectively improve the diagnostic efficiency of COVID-19. Maybe it can be further combined with the traditional features with depth features to improve the detection results.

## 4. Conclusion

In this paper, to identify COVID-19 chest X-ray images quickly and accurately, DPM module and DDS module are proposed. Based on these two modules, D2-CovidNet is designed with strong representation and low complexity. 4265 chest X-ray images of common pneumonia patients, 1575 normal chest X-ray images, and 412 chest X-ray images of COVID-19 patients are selected from two open-source datasets to train and evaluate the model. The experimental results show that D2-CovidNet has good performance, and its classification accuracy for images tested is 94.43%. Specifically, its accuracy, sensitivity, and specificity for COVID-19 are 98.97%, 94.12%, and 99.84%, respectively. Using D2-CovidNet to detect COVID-19 chest X-ray can effectively improve the diagnostic efficiency and help to detect and isolate patients in time, preventing the spread of 2019-nCoV. Although D2-CovidNet has good experimental results, it needs further clinical research and testing [32]. After further training and testing, D2-CovidNet is expected to be put into practical application in auxiliary diagnosis COVID-19.

## Figures and Tables

**Table 1:** Parameters of each layer of D2-CovidNet.

Layer (type)	Output shape	Params
Conv2d-1	[−1, 16, 224, 224]	464
DPM-2	[−1, 32, 112, 112]	1968
DPM-3	[−1, 48, 56, 56]	3888
DPM-4	[−1, 64, 28, 28]	6720
DPM-5	[−1, 80, 14, 14]	10320
DPM-6	[−1, 96, 7, 7]	14688
DW-7	[−1, 112, 7, 7]	12032
DDS-8	[−1, 16, 7, 7]	3056
DDS-9	[−1, 16, 7, 7]	3488
DDS-10	[−1, 16, 7, 7]	3920
DDS-11	[−1, 16, 7, 7]	4352
DDS-12	[−1, 16, 7, 7]	4784
DDS-13	[−1, 16, 7, 7]	5216
DDS-14	[−1, 16, 7, 7]	5648
DDS-15	[−1, 16, 7, 7]	6080
DDS-16	[−1, 16, 7, 7]	6512
Conv2d-17	[−1, 480, 1, 1]	124320
SoftMax-18	SoftMax-18	1443
Total params	218755	
Input size (MB)	0.57	

**Table 2:** Flops and parameters of other deep learning models and D2-CovidNet.

Model	Flops (million)	Params (million)
VGG19 [17]	18736.81	137.04
GoogleNet [18]	1434.21	5.32
ResNet50 [19]	3919.13	22.42
DenseNet121 [20]	2731.91	6.62
SqueezeNet1.0 [21]	702.71	0.73
MobileNet [22]	560.73	3.11
ShuffleNet [23]	142.02	0.91
MobileNetV2 [24]	311.13	2.13
ShuffleNetV2 [25]	144.72	1.22
D2-CovidNet	**71.27**	**0.20**

**Table 3:** Experimental platform configuration.

Attribute	Configuration information
Operating system	Ubuntu 16.04.5 LTS
CPU	Intel(R) Xeon(R) CPU E5-2670 v3 @ 2.30 GHz
GPU	GeForce GTX TITAN X
CUDNN	CUDNN 6.0.21
CUDA	CUDA 8.0.61
Frame	PyTorch
IDE	PyCharm
Language	Python

**Table 4:** The experimental results and model complexity.

Model	Average accuracy (%)					Flops (million)	Params (million)
	Epoch (1–50)	Epoch (51–100)	Epoch (101–150)	Epoch (151–200)	Epoch (191–200)		
MobileNetV2	75.5861	84.1087	83.7101	86.2726	89.8007	311.13	2.13
ShuffleNetV2	81.3605	86.5734	89.8614	89.5389	89.1169	144.72	1.22
D2-CovidNet	**85.4792**	**89.8542**	**90.7228**	**91.1304**	**92.0245**	**71.27**	**0.20**

**Table 5:** Values of criteria of experimented models.

Model	Accuracy (%)	Precision (%)	Sensitivity (%)	Specificity (%)	*F*1-score (%)
VGG19 [18]	93.11	**96.09**	92.93	96.47	93.02
GoogleNet [19]	92.56	95.29	91.56	95.78	92.06
ResNet50 [20]	93.53	96.01	93.15	96.53	93.34
DenseNet121 [21]	93.11	95.98	92.75	96.38	92.92
SqueezeNet1.0 [22]	67.91	45.83	50.51	64.16	57.93
MobileNet [23]	88.53	90.14	87.25	91.84	87.89
ShuffleNet [24]	87.02	90.08	86.17	92.31	86.59
D2-CovidNet	**94.43**	95.14	**94.02**	**96.61**	**95.30**

The best results are bolded.

**Table 6:** Values of criteria of models with different DPM and DDS configurations.

Model	Average accuracy (%)				
	Epoch (1–50)	Epoch (51–100)	Epoch (101–150)	Epoch (151–200)	Epoch (191–200)
F-DPM	83.5336	84.9918	86.0190	85.1278	85.3804
F-DDS	81.3605	86.5734	89.8614	89.5389	89.1169
D2-CovidNet	**85.4792**	**89.8542**	**90.7228**	**91.1304**	**92.0245**

The best results are bolded.

**Table 7:** Comparison of D2-CovidNet with other deep learning methods developed using X-ray images.

Method	Numbers of cases	Model	Accuracy (%)	Params (million)
Ioannis et al. [8]	224 COVID-19	Xception	92.85	33.00
	700 pneumonia			
	504 normal			

Wang et al. [9]	358 COVID-19	CovidNet	93.30	11.75
	5538 pneumonia			
	8066 normal			

Khan et al. [10]	284 COVID-19	CoroNet	**94.59**	33.00
	657 pneumonia			
	310 normal			

Ozturk et al. [11]	125 COVID-19	DarkCovidNet	87.02	1.10
	500 pneumonia			
	500 normal			

Our method	412 COVID-19	D2-CovidNet	94.43	**0.20**
	4265 pneumonia			
	1575 normal			

**Table 8:** Precision, sensitivity, and specificity of D2-CovidNet.

Class	Precision (%)	Sensitivity (%)	Specificity (%)
COVID-19	98.97	94.12	99.84
Normal	91.53	92.31	95.93
Pneumonia	94.91	95.64	94.05
Average	95.14	94.02	96.61

## Data Availability

The datasets are public datasets and can be found in References [26, 27].
